# Multidimensional interaction mechanisms and engineering applications of microalgae-fungal microorganism symbiotic systems

**DOI:** 10.3934/microbiol.2026013

**Published:** 2026-05-25

**Authors:** Yong Zhang, Yang Shen, Yining Gu, Yuan Yao, Meihan Liu, Yike He, Qiuzhen Wang

**Affiliations:** 1 Qinhuangdao Marine Center, the Ministry of Natural Resources, Qinhuangdao, 066000, China; 2 Marine Ecological Restoration and Smart Ocean Engineering Research Center of Hebei Province, Qinhuangdao, 066000, China; 3 The Eighth Geological Brigade, Hebei Geological Prospecting Bureau, Qinhuangdao, 066000, China; 4 Ocean College, Hebei Agricultural University, Qinhuangdao, 066000, China

**Keywords:** microalgae, filamentous fungi, yeasts, wastewater treatment, bio-flocculation, biorefinery

## Abstract

The symbiotic system of microalgae and fungal microorganisms, such as filamentous fungi, yeasts, and lichen-forming fungi, holds great potential in the fields of environmental remediation and bioresource development. Yet, it faces bottlenecks, including unclear symbiotic mechanisms, high energy consumption for biomass harvesting, poor adaptability to complex wastewater, and insufficient system integration. In this review, we systematically summarized the research progress on microalgae-fungi symbiotic systems in terms of germplasm resource exploration, multifaceted interaction mechanisms, and engineering applications. In particular, we focused on the core advantages of fungal pellet-assisted bioflocculation technology in overcoming the energy bottleneck of biomass harvesting, as well as its optimization strategies. The comprehensive performance of this system in wastewater purification, bioenergy accumulation, and high-value metabolite production was also evaluated. By integrating fundamental mechanisms with engineering application outcomes, we aimed to provide theoretical support and technical guidance for the construction of efficient, stable, and sustainable microalgae-fungi biorefinery platforms. Furthermore, we seek to facilitate the translation of this technology from laboratory research to industrial application.

## 1. Introduction

**Figure 1.**
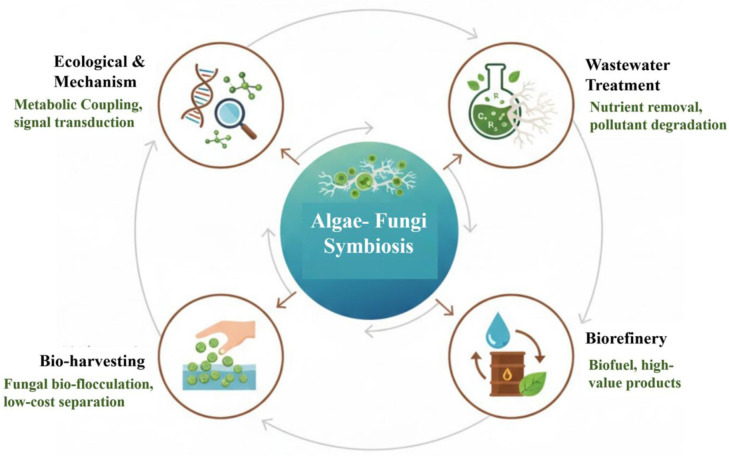
Application of the microalgal-fungi symbiotic system.

As key contributors to the global carbon cycle, microalgae exhibit considerable potential in addressing energy crises and environmental pollution owing to their high photosynthetic efficiency and metabolic products. However, monoculture systems of microalgae are often constrained by challenges such as poor environmental adaptability, imbalanced nutrient utilization, and high downstream harvesting costs [1],[2]. In recent years, inspired by natural lichen and symbiotic systems, co-culture systems integrating microalgae with eukaryotic microorganisms, such as filamentous fungi, yeasts, and lichen-forming fungi, have emerged as a research focus, commonly referred to as microalgae-eukaryote symbiosis (MES) [3]–[5]. These cross-kingdom symbiotic relationships not only enhance system stability through O₂/CO₂ complementarity and metabolic exchange [6],[7], but also enable fine regulation of physiological functions via the transmission of complex chemical signals, including indole-3-acetic acid (IAA) [6],[8]. At the application level, microalgae-fungi consortia have demonstrated synergistic effects in treating high-strength organic wastewater [9],[10], removing emerging contaminants such as antibiotics [11],[12], and producing biodiesel precursors alongside high-value functional compounds [13],[14] (Figure 1). Despite substantial progress achieved at the laboratory scale, deciphering the molecular mechanisms underlying symbiosis and realizing the transition from laboratory cultivation to engineering-scale implementation remain critical challenges that demand further investigation.

In this review, we systematically synthesize research advances in the field of microalgae-fungi symbiosis, with the aim of constructing a comprehensive “mechanism–process–application” knowledge framework. At the mechanistic level, we explore the multifaceted interaction mechanisms of microalgae-fungi symbiotic systems, including metabolic coupling, chemical communication, interfacial adhesion, and ecological evolution [3],[6],[8],[15]. Based on these mechanisms, we elucidate the underlying principles governing cross-kingdom microbial metabolic synergy and signal transduction. At the process level, we focus on fungal pellet-assisted bioflocculation harvesting technology, analyzing strategies for process parameter optimization, and the underlying micro-mechanisms [1],[2],[16]. Consequently, this review offers a technical pathway for overcoming the energy bottleneck associated with microalgae harvesting. At the application level, we systematically evaluate the purification performance of microalgae-fungi consortia in municipal wastewater, industrial effluents, and antibiotic-contaminated waters while exploring their resource recovery potential in bioenergy accumulation and the production of high-value functional compounds [13],[14],[17],[18]. In this review, we aim to provide a theoretical foundation and technical guidance for achieving deep wastewater purification, efficient CO₂ fixation, and the sustainable production of high-value bioproducts.

Although microalgae symbiotic systems have demonstrated considerable potential in the fields of environmental remediation and resource utilization, most research remains confined to the conventional microalgae-bacteria interaction paradigm. As summarized in Table 1, the microalgae-fungi systems (including filamentous fungi, yeasts, and lichen-forming fungi) offer distinct advantages over the classical microalgae-bacteria paradigm, particularly in biomass harvesting efficiency, system stability, and resource recovery potential. These advantages justify a paradigm shift in future research. Here, we achieve innovation and breakthroughs in the following three dimensions. First, we transcend the limitations of other research scopes by extending the perspective to the more complex microalgae-fungal microorganism systems. We systematically encompass fungal partners, such as filamentous fungi, yeasts, and lichen-forming fungi [3],[4],[19],[20]. Second, through in-depth analysis of the regulatory role of diffusible signaling molecules (e.g., IAA) in non-contact co-culture systems, combined with transcriptomic and metabolomic evidence, we elucidate the underlying mechanisms governing pollutant degradation and directed biomass accumulation [11],[21],[22]. Third, adopting an application-oriented approach, we focus on the coupling logic between one-step co-culture and self-flocculation harvesting technology. By comparing the performance differences of microalgae-fungi combinations in typical industrial scenarios such as starch wastewater, swine wastewater, and oilfield polymer-flooding produced water, we prospectively evaluate the industrial feasibility of an integrated “pollution control-harvesting-valorization” process [9],[10],[23],[24]. Through this multidimensional integrative analysis, we aim to provide novel theoretical support and technical guidance for the construction of efficient and stable next-generation microalgae-fungi symbiotic platforms.

**Table 1.** Comparison of microalgae-based symbiotic systems: bacteria, filamentous fungi, yeasts, and lichen-forming fungi.

Comparison dimension	Microalgae–bacteria system	Microalgae–filamentous fungi system	Microalgae–yeast system	Artificial lichen-like structure
Symbiotic mechanism	O₂/CO₂ complementarity, metabolic exchange, quorum sensing	Hyphal-assisted bioflocculation, chemical signal transduction (e.g., IAA), metabolic coupling	Metabolic complementarity, adhesive interactions	Fungal hyphae provide a physical scaffold, with microalgae embedded to form a stable structure
Biomass harvesting efficiency	Low, requiring centrifugation/flocculation assistance	High (>95%), achieved through hyphal self-flocculation	Moderate, partial flocculation capability	High, with hyphal network immobilizing microalgae
Pollutant removal	Good nutrient removal	Treatment of high-concentration organic wastewater, antibiotics, and other emerging contaminants	Moderate, suitable for specific wastewaters	Long-term stability, strong stress tolerance
System stability	Moderate, susceptible to environmental fluctuations	High, with hyphae providing physical support and stress tolerance	Moderate	Very high, natural symbiotic structure
Resource recovery potential	Biomass, methane	Biodiesel precursors, high-value compounds, organic substrates	Ethanol, high-value metabolites	Long-term carbon fixation, bioactive compounds
Process complexity	Relatively simple, well-established in research	Moderate, requiring optimization of strain pairing and cultivation conditions	Simple	Relatively high, requiring artificial construction of stable symbionts
Industrial maturity	High (widely applied)	Laboratory stage, requiring pilot-scale validation	Moderate (relatively mature for ethanol production)	Low, mainly at the conceptual exploration stage
Key advantages	well-studied, easy to operate	Self-flocculation harvesting, anti-contamination, multifunctional integration	Ethanol fermentation synergy, rich metabolite profile	Adaptation to extreme environments, long-term stability
Key limitations	High harvesting energy consumption, poor stress tolerance	Lack of scale-up validation, risk of fungal contamination	Limited flocculation efficiency, strain compatibility issues	Difficulty in artificial construction, unclear mechanisms

## 2. Screening of indigenous strains and fungal pellet-based harvesting technology

### 2.1. Isolation, purification, and medium optimization of strains from extreme environments

The isolation and optimization of germplasm resources represent the initial step toward the industrial application of microalgae-fungi technology. In natural aquatic environments, fungal communities exhibit pronounced dynamic succession patterns, providing ecological insights for the design of artificial symbiotic systems. The exploration of indigenous strains from extreme environments, such as saline-alkaline and arid habitats, serves as the foundation for constructing robust symbiotic systems. Researchers have isolated and purified *Scenedesmus bajacalifornicus* and *Desmodesmus abundans* from saline–alkaline waters [25]. In addition, the key components of their culture media were systematically optimized, significantly improving the survival advantages of these microalgae under saline–alkaline conditions [25].

Toxic algal hosts harbor highly conserved and functionally diverse core endophytic fungal taxa, which may play important roles in nutrient acquisition and stress tolerance, suggesting the existence of stable intrinsic associations between specific algal species and their fungal partners [26]. Therefore, when exploring indigenous microalgae from habitats such as saline-alkaline and arid environments, their naturally associated fungal partners represent an equally indispensable germplasm resource for constructing robust symbiotic systems. Furthermore, understanding the natural associations between these special strains and native fungal communities facilitates the rapid establishment of artificial symbiotic systems under resource-limited conditions. This de novo screening and optimization process ensures the ecological adaptability of the system.

### 2.2. Fungal pellet-based bioflocculation harvesting technology and its efficacy

Low-cost harvesting of microalgal biomass represents a critical bottleneck in the production chain, and fungal pellet-based bioflocculation offers a transformative solution. Precise control of process parameters directly determines the performance output of microalgae-fungi systems. Under optimal process conditions for key parameters such as flocculation mode, temperature, agitation speed, and initial pH, *Aspergillus niger* pellets achieve a flocculation efficiency of 99.4% for *Scenedesmus* sp. [1]. When optimizing the bioflocculation conditions of *Chlorella vulgaris* using *Aspergillus niger* pellets, the interactions among bioflocculant dosage, cationic inducer concentration, and agitation speed significantly influence harvesting efficiency [27]. Under optimal conditions, the harvesting efficiency reaches 98% [27]. In optimizing the bioflocculation conditions of oleaginous microalgae *Chlorella* sp. using the lipid-rich, cellulose-degrading fungus *Aspergillus terreus*, microalgal age, pH, cell density, flocculant ratio, and contact time are identified as key factors affecting harvesting efficiency, with a maximum harvesting efficiency of 97.6% achieved under optimal conditions [28]. Comparative studies revealed that pre-cultured fungal pellets exhibit significantly superior flocculation speed and stability compared to co-culture modes involving direct inoculation of fungal spores, effectively circumventing the lag phase associated with spore germination [1].

The high efficiency and stability of fungal pellet-based bioflocculation can be attributed to the expansion and deepening of fungal species and their modes of action. On one hand, *Aspergillus oryzae*, which is FDA-approved, can be directly used as a flocculant in the form of mycelial pellets, achieving a flocculation efficiency of over 95% across a broad pH range of 4.0–9.0, offering advantages in terms of safety, broad-spectrum applicability, and rapid response [16]. On the other hand, *Aspergillus fumigatus*-assisted flocculation reduces the processing time from tens of hours to within 3 hours, overcoming the speed bottleneck, and is applicable to a variety of microalgal species and wastewater culture systems [2]. However, it is worth noting that *Aspergillus fumigatus* is a potentially pathogenic fungus, and its use limits the application of harvested microalgae in food or feed products. Both types of fungal pellets are easy to separate and do not cause secondary pollution, further solidifying the feasibility of bioflocculation as a low-cost, low-energy harvesting strategy for microalgae.

### 2.3. Safety considerations and microalgae-dependent performance of fungal bioflocculation

Increasing attention has been given to edible and safe fungi for microalgae harvesting to ensure the suitability of the harvested biomass for human or animal consumption. For instance, *Ganoderma lucidum*, a well-known edible medicinal mushroom, has been investigated for bioflocculating *Chlorella* sp., achieving a harvesting efficiency of 39.7% under optimized conditions (pH 9, 23 °C, 100 rpm mixing speed) [30]. Another edible fungus, *Pleurotus ostreatus*, demonstrates a harvesting efficiency of 74% for *Chlorella sorokiniana* at pH 4.5 with extended harvesting time [31] and achieves 62–75% harvesting efficiency for *Euglena gracilis* [32]. Although these edible fungal species generally exhibit lower flocculation efficiencies than *Aspergillus niger* (99.4%) and *Aspergillus oryzae* (>95%) (Table 2), they offer a significant advantage in terms of biosafety. This characteristic makes them particularly suitable for harvesting food-grade microalgae intended for nutraceutical, feed, and human consumption applications.

It should be noted that the efficiency of fungal bioflocculation is significantly influenced by the surface properties of different microalgae. Consequently, not all microalgae are equally suitable for this strategy. *Euglena gracilis*, for instance, lacks a rigid cell wall and possesses flagella-driven motility, which makes it inherently difficult to harvest using conventional methods. As demonstrated above, fungal-assisted flocculation achieves a harvesting efficiency of only 62–75% for *Euglena gracilis* [32]. This efficiency is considerably lower than the >95% typically observed for cell-walled microalgae, such as *Chlorella* and *Scenedesmus*, under optimized conditions. Therefore, when designing fungal-based harvesting processes, key microalgal traits, including the presence or absence of a cell wall, motility, surface charge and cell size, must be carefully considered. In addition, fungal flocculants should be selected on a case-by-case basis.

Furthermore, the choice between safe and unsafe fungal flocculants depends on the downstream application of the harvested microalgal biomass. For applications such as biofuel production or wastewater treatment, where product safety is of lesser concern, non-edible or potentially pathogenic fungi, including *Aspergillus fumigatus* and *Aspergillus niger*, may be preferable. Their advantages lie in their superior flocculation efficiency and greater environmental robustness. However, for the production of high-value microalgal biomass intended for food, feed, or pharmaceutical applications, the use of edible fungi is strongly recommended. Species such as *Aspergillus oryzae*, *Ganoderma lucidum*, *Pleurotus*, and *Lentinus* are preferred because of their superior biosafety, despite their relatively lower flocculation efficiencies.

**Table 2.** Comparison of flocculation performance across fungus-microalgae combinations.

Fungal species	Algal species	Key parameters for optimization	Flocculation efficiency	Ref.
*Aspergillus niger*	*Scenedesmus* sp.	Flocculation method: Co-culture→pre-culture; Temperature: 20 °C/40 °C→30 °C; Agitation rate: 0/240 rpm→160 rpm; Initial pH: 3.0~9.0→8.0	Up to 99.4%	[1]
*Aspergillus fumigatus*	*Aspergillus fumigatus*	Fungal culture time: 72 h→24 h; Temperature: 28 °C→38 °C; Algae-to-fungi ratio: 1:3/1:7/1:9→1:5; Agitation rate: 120/150 rpm→80/100 rpm	Increased from 76% to 99%	[2]
*Aspergillus oryzae*	*Microcystis aeruginosa*	Fungal culture time: 3 to 8 days→6 days; Dosage: 6.5g /L→11g /L; Agitation rate: 50/150/200 rpm→100 rpm; Temperature: 15/20/30 °C→25 °C; pH：4.0~9.0	Up to 99.06%	[16]
*Aspergillus niger*	*Chlorella vulgaris*	Bioflocculant dose: 2.5%/4.5% w/v→3.5% w/v; Cationic inducers: 0.05/0.07 mM→0.06 mM; Agitation rate: 150/170 rpm→160 rpm; Initial pH 8.0	Increased from approximately 45% to 98%	[27]
*Aspergillus terreus* MD1	*Chlorella* sp.	Contact time: 1 h→24 h; pH: 7→6; Algae-to-fungi ratio: 10%→30%; Algal age:1 week→3 weeks; Algal density (OD₆₈₀): 1→3	Increased from 82.7% to 97.6%	[28]
*Aspergillus niger*	*Chlorella vulgaris*	pH：5~9→Natural pH7.0; Ca²⁺：0→0.1 g/L; Mg²⁺：0→0.5 g/L; NaCl：1→30 g/L	Increased from 83% to 97.75%	[29]
*Ganoderma lucidum*	*Chlorella* sp.	pH 9; Reaction temperature: 23 °C; Agitation rate: 100 rpm; 40 mg/mL (wet weight) fungal pellets	Reached as high as 39.7%	[30]
*Pleurotus ostreatus*	*Chlorella sorokiniana*	the maximum harvesting efficiency of 74%; pH 4.5; Large-sized fungal pellets (formed after 9 days) and extended harvesting periods (24 h)	Maximum harvesting efficiency of 74%	[31]
*Ganoderma lucidum*	*Euglena gracilis*	Fungi to algae ratio of 1:2; Harvested at 1 h	Maximum harvesting efficiency of 62%	[32]
*Pleurotus ostreatus*	*Euglena gracilis*	Fungi to algae ratio of 1:1; Harvested at 2 h	Maximum harvesting efficiency of 70%	[32]
*Penicillium restrictum*	*Euglena gracilis*	Fungi to algae ratio of 1:2; Harvested at 2 h	Maximum harvesting efficiency of 75%	[32]

## 3. Multifaceted mechanisms of microalgae-fungi interactions: From metabolic complementation to chemical communication and ecological evolution

### 3.1. Metabolic coupling of respiration and photosynthesis and its role in stability maintenance

The mutualistic symbiosis between microalgae and fungal microorganisms, including filamentous fungi and lichen-forming fungi, is primarily characterized by a balanced exchange of O₂ and CO₂. Through photosynthesis, microalgae supply oxygen to heterotrophic eukaryotes, thereby alleviating the oxygen limitation commonly encountered in high-density cultivation systems. In turn, fungal microorganisms release CO₂ through respiration, providing an essential carbon source for microalgal carbon assimilation. Additionally, acidic metabolites produced by fungi can neutralize the alkaline substances generated during microalgal photosynthesis, thereby contributing to pH homeostasis within the symbiotic system [3]. This metabolic complementarity is particularly important in extreme environments. In alpine lichen systems, for example, fungal hyphae provide physical protection to microalgae against intense ultraviolet radiation and drought stress, highlighting the remarkable adaptability of these symbiotic associations during ecological succession [4],[7]. Overall, metabolic balance constitutes the fundamental basis that enables microalgae–fungi symbiotic systems to maintain long-term stability and resilience under fluctuating environmental conditions.

### 3.2. Precise chemical communication mediated by diffusible signaling molecules

In recent years, research on microalgae-fungi interactions has shifted from simple material exchange to the complex regulation mediated by chemical signaling. Using non-contact dialysis co-culture systems, studies have demonstrated that certain fungi, such as *Clonostachys rosea*, can significantly stimulate microalgal growth through the release of diffusible metabolites, including IAA and tryptophan, even in the absence of direct physical contact with microalgal cells [6]. Transcriptomic analyses further revealed that these signaling molecules induce the upregulation of genes associated with photosynthesis and amino acid metabolism in microalgae, thereby enhancing electron transport efficiency and promoting substrate degradation [6],[8]. This intricate communication network not only regulates population dynamics, but also enables precise modulation of physiological and metabolic fluxes. Consequently, elucidating the spatiotemporal distribution and perception mechanisms of these signaling molecules is essential for understanding the co-evolutionary dynamics underlying microalgae-fungi interactions.

### 3.3. Physical adhesion at the symbiotic interface and the bridging mechanism of extracellular polymeric substances (EPS)

Tight physical association serves as a prerequisite for functional integration and engineering application of microalgae-fungi symbiotic systems. The interaction between the three-dimensional framework provided by fungal hyphae and microalgal cells is synergistically driven by Zeta potential, hydrophobic interactions, and surface functional groups [3],[33]. Notably, the EPS secreted by fungi are rich in proteins, polysaccharides, and protein-like compounds, such as tyrosine and tryptophan. These components can interact with amino, hydroxyl, and amide groups on microalgal cell surfaces through electrostatic attraction, thereby promoting crosslinking and the formation of stable bioball structures [3],[7]. This physical embedding not only optimizes light utilization efficiency but also enhances the system's resistance to fluid shear forces. Physical attachment and chemical adhesion together constitute the homeostatic boundary of microalgae-fungi symbiotic systems.

### 3.4. Mutualistic evolution of fungi and microalgae and their metabolic adaptability in extreme environments

Lichens, as the quintessential natural model of microalgae-fungi symbiosis, provide important insights for the artificial construction of stable symbiotic systems. Within lichen symbioses, fungi not only supply nutrients to their associated green algae but also promote mutualistic co-evolution through complex biological interactions [4],[7]. Such cooperative relationships enable the symbiotic consortium to thrive in nutrient-limited and extreme environments. Moreover, these associations exhibit remarkable physiological flexibility under fluctuating environmental conditions. For example, photosynthetic metabolism is enhanced under adequate water availability, whereas the system can transition into a dormant protective state during drought stress. This evolutionary strategy observed in nature offers valuable theoretical guidance for the laboratory-based design of efficient and resilient symbiotic systems through artificial intervention. Overall, lichen-like symbiotic architectures exemplify the core ecological and adaptive advantages of cross-kingdom cooperation between microalgae and fungi in extreme ecological niches.

### 3.5. Negative interactions of fungi on microalgae: from algal bloom control to pollution management

Complex relationships of predation, infection, and competition also exist between fungi and microalgae, which hold significant value in the field of algal bloom control. For instance, certain chytrid fungi can selectively infect harmful algal bloom species, such as dinoflagellates, and induce algal cell lysis through specialized infection mechanisms [15],[33],[34]. White rot fungi effectively disrupt the metabolic networks of harmful algal cells through the production of extracellular enzymes or reactive oxygen species, achieving efficient algal control [14]. This "turn enemies into friends" approach not only safeguards aquaculture safety, but also enables the conversion of harmful algae into utilizable organic substrates [14],[35]. Furthermore, bifunctional fungi can achieve dual intervention in water eutrophication by balancing denitrification performance with algicidal activity [8]. Studies on these negative interactions enrich our understanding of the complexity of microalgae-fungi ecological networks. Elucidating the pathogenic mechanisms of fungi against microalgae holds important practical significance for the development of efficient and environmentally friendly biological algicides [8],[36].

Moreover, elucidating antagonistic interactions reveals critical vulnerabilities in symbiotic systems, enabling the regulation of negative interactions to avert collapse and bolster system resilience. In open cultivation systems, microalgae often face fungal contaminants. The interactions between fungi and microalgae are complex, potentially manifesting either as pathogenic parasitism or mutualistic symbiosis through co-culture, depending on the strain combination and cultivation conditions [33]. Studies have shown that these dynamic interactions not only affect microalgal growth but also profoundly alter the metabolic profiles within the symbiotic system, leading to unique metabolic effects such as those observed during the co-culture of *Galdieria sulphuraria* and *Penicillium citrinum* [37]. This dynamic balance based on defense and response ensures the survival of the symbiotic system under complex biological backgrounds. Therefore, a deeper understanding of interaction mechanisms within microecological environments will facilitate the development of more robust and resilient industrial production systems.

## 4. Multifunctional applications of microalgae-fungi symbiotic systems in complex wastewater treatment

### 4.1. Synergistic nutrient removal in municipal and livestock wastewater treatment

In the treatment of municipal sewage and livestock wastewater, microalgae-fungi systems exhibit significantly higher nutrient removal efficiencies compared to monoculture modes. Microalgae efficiently assimilate inorganic nitrogen and phosphorus through uptake, while eukaryotic microorganisms eliminate metabolic obstacles by degrading macromolecular organic matter [10],[38]. For instance, in swine wastewater treatment, microalgae-fungi consortia achieve high COD removal rates while utilizing EPS-mediated aggregate structures to enhance settleability [10]. Furthermore, through biogranulation technology, this system can capture CO₂ from biogas while treating biogas slurry, thereby increasing methane content and achieving energy upgrading [38]. Addressing the dual challenges of high CO₂ content in biogas and high nutrient pollution in biogas slurry, the construction of a fungi-assisted microalgae granulation system enables simultaneous biogas slurry purification and biogas upgrading [39]. The development of a fungi-algae self-flocculation system has demonstrated significant removal efficiencies for pollutants such as nitrogen, phosphorus, and COD in filter mud leachate from the sugar industry [40]. Besides, this enables treated wastewater to be recycled and reused while achieving high biomass yields [40]. Therefore, microalgae-fungi synergy not only enables water purification, but also achieves the integration of pollution control and resource recovery.

### 4.2. Metabolic complementation and substrate broadening in industrial wastewater treatment

When confronted with complex industrial wastewaters, microalgae-fungi systems exhibit a unique capacity for substrate broadening. In the treatment of alkali pretreatment liquor (APL) with extremely high lignin content, *Cunninghamella echinulata* first degrades macromolecular lignin using its robust extracellular enzyme system, followed by assimilation of the resulting small organic molecules by *Chlorella sorokiniana*, resulting in a 2.75-fold increase in total biomass [21]. This metabolic complementation, characterized by fungal degradation of macromolecules and microalgal assimilation of small molecules, enables the system to directly treat high-concentration APL wastewater without dilution. Microalgae-fungi systems could thereby effectively address the challenge that conventional biological methods face in directly utilizing organic carbon sources from industrial waste streams [21].

A similar synergistic strategy has also been validated in starch wastewater treatment. A one-step co-culture system of *Aspergillus oryzae* and *Chlorella pyrenoidosa* efficiently treats starch wastewater while producing high-value biomass [9]. In this system, *Aspergillus oryzae* and *Chlorella pyrenoidosa* act synergistically, significantly enhancing the removal efficiencies of chemical oxygen demand (COD), total nitrogen (TN), and total phosphorus (TP), with the final biomass concentration in the co-culture system exceeding that achieved in monocultures. Notably, during cultivation, free microalgal cells are progressively adsorbed onto and encapsulated within fungal pellets, enabling complete biomass harvesting within 72 hours [9]. This process effectively addresses the challenge of the high costs associated with microalgal biomass recovery. Through synergistic multi-enzyme systems or charge neutralization, microalgae-fungi consortia can establish an efficient organic matter conversion network.

### 4.3. Targeted degradation and nanomodulation of high-strength industrial wastewater

For the treatment of refractory polyacrylamide (PAM) and supersaturated inorganic carbon in oilfield polymer-flooding produced water, microalgae-fungi consortia offer an innovative remediation strategy. A constructed *Fusarium* sp. - *Chlorella* sp. consortium achieves a PAM degradation rate of 80.93% through the secretion of extracellular amidases by the fungus to degrade side and major chains of PAM, complemented by the fixation of inorganic carbon by microalgae [23]. Furthermore, the addition of co-metabolic nitrogen sources such as urea can further activate the degradation activity of the system, with no secondary toxic byproducts generated during the treatment process [23]. This targeted remediation capability directed at specific chemical structures expands the boundaries of biological treatment technologies.

In recent years, researchers employing nanomaterials to regulate the performance of microalgae-fungi consortia have also provided new insights for wastewater treatment. Appropriate addition of carboxylated multi-walled carbon nanotubes (1.5 mg/L) significantly promotes the growth and photosynthesis of the consortia, achieving efficient removal of COD, total nitrogen, total phosphorus, and tetracycline hydrochloride during simulated digestate treatment [41]. This strategy optimizes consortium functionality through a concentration-dependent “low-promotion, high-inhibition” effect, offering a referable regulatory approach for the efficient treatment of complex wastewater. Furthermore, in the presence of nanocellulose, the binding strength between white rot fungi and microalgae is significantly enhanced, effectively addressing the issue of detachment under high-shear environments [42]. Moreover, cations of different valences exert inductive effects on the co-pelletization process, with ion concentration promoting microalgae-fungi adsorption by modulating electrostatic repulsion on cell surfaces [29]. This interdisciplinary research at the interface of materials science and colloid chemistry provides new tools for optimizing wastewater treatment processes.

### 4.4. Process parameter optimization drives enhanced performance of microalgae-fungi wastewater treatment

**Figure 2.**
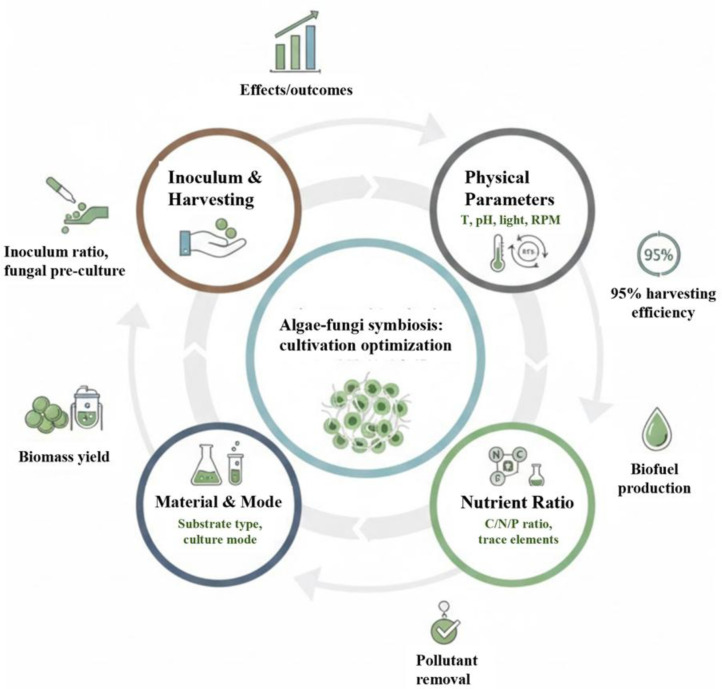
Optimization of microalgae-fungi culture process and key influencing factors. Harvesting efficiency above 95% are typically observed for cell-walled microalgae such as *Chlorella* sp. and *Scenedesmus* sp. under optimized conditions.

Wastewater treatment efficiency is highly dependent on the optimization of process parameters such as microalgae-fungi ratio, light conditions, and agitation speed (Figure 2). In the treatment of biogas slurry, a red-to-blue light mixing ratio of 5:5 combined with a microalgae-fungi inoculation ratio of 1:10 achieves removal rates of up to 90% for COD and total nitrogen [39]. Furthermore, depending on the characteristics of different wastewaters, such as those containing signaling factors like synthetic strigolactone (GR24), the response of microalgae-fungi systems exhibits pronounced species specificity [38].

In terms of optimizing flocculation and sedimentation parameters, studies have demonstrated that a self-flocculation system based on *Aspergillus niger* and *Chaetomium gracile* can achieve a gravity-settling flocculation efficiency of 99.6% within 4 hours when the fungal pellet diameter is maintained at 10 mm and the temperature is controlled at 30 °C [40]. Furthermore, the addition of Ca²⁺ has been shown to further enhance flocculation performance [40]. Following treatment, the wastewater can be recycled and reused, achieving a biomass yield of 5.75 g/L, thereby realizing the dual objectives of efficient purification and resource recovery [40]. Through precise regulation of these key factors, metabolic flux can be artificially directed toward specific purification pathways. Therefore, integrated optimization of process parameters is essential for achieving stable wastewater treatment and efficient output.

### 4.5. Application of microalgae-fungi consortia in heavy metal and specialty wastewater remediation

The three core mechanisms underlying fungi-assisted microalgae harvesting, namely surface charge alteration, hydrophobic interactions, and microbial surface component interactions, provide directions for optimizing key parameters such as the fungi-algae ratio, pH, and carbon source [3],[43]. Building on these synergistic mechanisms, microalgae-fungi consortia have demonstrated efficient removal of organic carbon, nitrogen, phosphorus and heavy metals (e.g., cadmium, copper and arsenic) in diverse wastewater treatment applications, including livestock wastewater, food-processing wastewater and biogas slurry. The potential for utilizing the resulting biomass in applications such as animal feed and biofuels is also being progressively explored [43].

Microalgae-fungi systems exhibit outstanding performance in the removal of heavy metals and pharmaceutical pollutants. Fungal-microalgal pellets constructed with filamentous fungi such as *Aspergillus flavus* demonstrate exceptionally high adsorption capacity for Cu(II) in aqueous environments due to their large surface area and abundant functional groups [44]. In the treatment of petroleum extraction produced water, microalgae-fungi systems significantly reduce water toxicity through physical entrapment and biotransformation [45]. This multi-stage entrapment and purification mechanism ensures the reliability of effluent quality. In summary, microalgae-fungi systems occupy an irreplaceable position in the remediation of industrial specialty wastewater and the prevention and control of heavy metal pollution.

### 4.6. System integration of microalgae-fungi symbiosis technology and its application in green water treatment

Microalgae-fungi symbiosis technology is progressively advancing from single-component research toward system integration, with the aim of constructing sustainable bioremediation platforms. The introduction of reinforcing materials such as carbon nanotubes can significantly enhance the formation of microalgae-fungi consortia and their metabolic stability in complex water bodies [41]. Furthermore, for highly challenging wastewaters such as filter mud leachate from the sugar industry, microalgae-fungi self-flocculation systems have demonstrated excellent industrial applicability [40].

In the treatment of food processing wastewater, a co-culture system of *Chlorella sorokiniana* and *Aspergillus oryzae* achieves integrated microalgae harvesting, wastewater purification, and pellet recovery. Under optimal conditions, the harvesting efficiency reaches 98% within 6 hours, while the removal efficiencies for total nitrogen and total phosphorus exceed 90% [17]. Notably, the fungal pellets in this system are reusable and eliminate the biomass contamination issues commonly associated with chemical flocculants [17]. Consequently, this approach provides a sustainable solution for green water treatment that is well aligned with the principles of the circular bioeconomy. This integrated approach not only broadens the spectrum of pollutant removal, but also reduces treatment costs through biomass recovery. In summary, microalgae-fungi symbiosis technology represents a key driver for the future transformation of green water treatment processes toward resource recovery.

## 5. High-value biomass directed accumulation and conversion for biorefinery

### 5.1. Synergistic production of lipids and optimization of fatty acid composition

Co-culture of microalgae with fungi offers a new approach for efficient production and quality regulation of biofuels. Through rational strain pairing and optimized nutritional design, co-culture systems not only enhance biomass productivity but also improve fatty acid composition [14]. This strategy effectively alleviates the poor oxidative stability of biodiesel associated with the excessively high proportion of polyunsaturated fatty acids (PUFAs) typically observed in monoculture microalgal systems [14]. Nutritional conditions significantly influence lipid accumulation. Glucose supplementation alone increases total lipid content, whereas the combination of glucose and peptone is most favorable for biomass enhancement [20]. *Nannochloropsis salina* and *Rhodosporidium toruloides* achieve metabolic synergy without the need for complex regulation, enabling simultaneous accumulation of lipids and carotenoids [13].

Moreover, co-culture of microalgae with filamentous fungi can significantly reduce the proportion of PUFAs in microalgal lipids, rendering the biomass a high-quality feedstock for biodiesel production [14]. Microalgae-fungi co-culture exerts synergistic enhancing effects on metabolites such as lipids and proteins [20]. An appropriate inoculation ratio contributes to maintaining a stable population structure [20]. Co-cultivation of microalgae and yeasts enables stable coexistence under suboptimal conditions. In this system, microalgal metabolism can alleviate the pH decline induced by yeast growth while promoting carotenoid accumulation [13]. Microalgae-fungi co-culture offers significant advantages in increasing lipid yield, optimizing fatty acid composition, and simplifying operational procedures through metabolic synergy and nutritional compatibility. Future efforts focusing on optimizing nutritional ratios, developing waste-derived alternative nutrient sources, and screening compatible strains are expected to advance this technology toward large-scale and sustainable development.

### 5.2. Synergistic removal and bioremediation of antibiotic contamination

Microalgae-fungi symbionts exhibit significant advantages in antibiotic treatment. The *Chlorella* sp.-*Aspergillus caespitosus* symbiont achieves a tetracycline removal rate of 93.00% and a biomass harvesting efficiency of 92.69% [11]. The underlying metabolic mechanism involves enhanced secretion of proteins and polysaccharides within EPS, which activates intracellular amino acid and nucleotide metabolic pathways in response to antibiotic stress [11]. This coordinated response enables the simultaneous achievement of pollutant removal and biomass accumulation. Furthermore, this system exhibits a lower frequency of horizontal gene transfer, reducing the risk of antibiotic resistance gene dissemination and offering enhanced biosafety [11]. Similarly, sub-inhibitory concentrations of erythromycin promote the formation of co-flocculates between *Penicillium* sp. and microalgal species [12]. This interaction results in a 2.4- to 14.5-fold increase in biomass relative to monoculture microalgal systems, together with an 11.2-fold enhancement in CO₂ sequestration capacity and a protein content of up to 34% [12]. Transcriptomic analysis further revealed the upregulation of genes associated with lipid synthesis and extracellular polysaccharides, and the co-flocculates protect microalgae from erythromycin-mediated chlorosis damage [12]. Microalgae-fungi symbiotic systems achieve efficient antibiotic removal and synergistic biomass recovery through the coordinated activation of metabolic pathways, enhanced secretion of EPS, and the formation of co-flocculated structures. This provides an important foundation for the treatment of antibiotic-contaminated wastewater while ensuring the safety and quality of the harvested biomass.

### 5.3. Application potential of microalgae-fungi symbiotic systems in functional ingredient production

Microalgae-fungi systems serve as efficient platforms for the production of natural pigments and antioxidant compounds. Under specific environmental stresses and in mixed culture media, the ability of yeasts to accumulate astaxanthin and carotenoids is significantly enhanced with the assistance of microalgae [13]. Metabolomic analysis enables the identification of key metabolite nodes driving product synthesis during symbiosis, thereby facilitating the directed accumulation of target compounds [22]. Studies have demonstrated that optimizing macronutrient combinations, such as glucose and peptone, together with appropriate inoculation ratios (yeast:microalgae = 1:4), can significantly enhance biomass production and promote the accumulation of high-value products, including lipids, proteins and carbohydrates in co-culture systems [20].

Furthermore, co-culture of marine microalgae and fungi can induce the production of specialized metabolites such as β-carboline alkaloids and sydowinin series compounds, which exhibit superior bioactivity compared to those produced in monocultures [35]. This offers new sources for the development of functional food ingredients and cosmetic additives. Additionally, this “one-pot, multi-product” approach greatly enriches product diversity and enhances the full-chain value of biorefinery. Thus, microalgae-fungi symbiotic systems hold broad prospects for the extraction of functional food ingredients and cosmetic additives.

### 5.4. Resource recycling and biomass conversion based on waste substrates

The industrial appeal of microalgae-fungi symbiotic systems lies in their efficient utilization of low-cost industrial wastes. Using sugarcane molasses and molasses wastewater as substrates, fungal mycelia serve as natural carriers for microalgae harvesting, achieving a closed loop of biomass accumulation and wastewater treatment [46],[14]. In this one-step process, the system not only achieves high biomass yield but also significantly optimizes the fatty acid profile of the biomass, rendering it more suitable for industrial applications [46],[24]. This “treating waste with waste and turning waste into valuable resources” approach substantially reduces raw material costs. The microalgae-fungi conversion technology based on low-cost substrates represents a crucial component for achieving circular economy goals.

### 5.5. Economic viability and sustainability assessment of microalgae-fungi symbiosis technology

The ultimate assessment of microalgae-fungi symbiosis technology must return to considerations of economic viability and sustainability. Flocculation assisted by *Aspergillus niger* pellets not only significantly reduces the energy consumption associated with microalgae harvesting, but also yields biomass that is more readily converted into biofuel through thermochemical processes [47],[24]. With the aid of inducing factors such as erythromycin, the symbiotic system can achieve increases in biomass and carbon fixation even under organic-free culture conditions, demonstrating substantial potential for carbon emission reduction [12]. These optimization strategies render the technology more attractive along the commercialization pathway. As a sustainable green manufacturing platform, the comprehensive benefits of microalgae-fungi symbiotic systems have been robustly supported by studies.

Moreover, despite the promising laboratory results, several bottlenecks hinder the industrial application of microalgae-fungi symbiotic systems. First, most researchers use batch-mode shake flasks, whereas continuous operation in large-scale reactors remains unexplored. Second, although open pond cultivation is economically attractive, the introduction of exogenous eukaryotic microorganisms poses a serious risk of biological contamination, for which effective control strategies are unavailable. Third, the production cost of fungal biomass using laboratory-grade media is prohibitively high, and low-cost substrates need to be developed. Finally, pilot-scale validation is critically absent. Accordingly, greater attention should be devoted to these engineering considerations before the industrial viability of the system can be established.

## 6. Conclusions

Based on the multifaceted synergistic mechanisms of metabolic complementation, chemical communication and interfacial adhesion, microalgae-fungal microorganism symbiotic systems exhibit unique scientific and engineering value in the fields of environmental remediation and high-value biorefinery. Fungal pellet-assisted bioflocculation technology can achieve harvesting efficiencies exceeding 98% through the coordinated effects of electrostatic attraction, physical adsorption, and chemical adsorption, coupled with process parameter optimization. Moreover, fungal pellets offer distinct advantages, including easy separation and the absence of secondary pollution. Microalgae-fungi symbiosis not only maintains metabolic homeostasis through O₂/CO₂ exchange but also mediates cross-kingdom chemical communication via signaling molecules such as IAA. In addition, fungal hyphae and EPS establish stable interfacial structures through physical adhesion. In wastewater treatment applications, microalgae-fungi systems exploit the metabolic complementarity between fungal degradation of macromolecular pollutants and microalgal assimilation of small molecules, thereby enabling broad-spectrum adaptability from municipal sewage to high-strength industrial wastewater. At the biorefinery level, co-culture systems can effectively optimize the fatty acid composition of biodiesel while facilitating the targeted production of high-value functional compounds, including carotenoids and β-carboline alkaloids. As such, researchers should prioritize elucidating molecular regulatory mechanisms, identifying novel microalgae-fungi consortia, designing scalable bioreactor systems, and conducting life-cycle and techno-economic assessments. These efforts will accelerate the efficient transition of microalgae-fungi symbiosis technologies from laboratory-scale studies to industrial-scale applications.

## Use of AI tools declaration

The authors utilized AI-assisted editing tools DeepSeek for the purpose of improving language fluency and readability, as well as figure conception. The final content was reviewed and verified by the authors.
